# LIDAR Developments at Clermont-Ferrand—France for Atmospheric Observation

**DOI:** 10.3390/s150203041

**Published:** 2015-01-29

**Authors:** Patrick Fréville, Nadège Montoux, Jean-Luc Baray, Aurélien Chauvigné, François Réveret, Maxime Hervo, Davide Dionisi, Guillaume Payen, Karine Sellegri

**Affiliations:** 1 Observatoire de Physique du Globe de Clermont-Ferrand, Université Blaise Pascal, 24 av. des Landais, BP80026, Aubière Cedex 63171, France; E-Mail: J.L.Baray@opgc.fr; 2 Laboratoire de Météorologie Physique–UMR 6016, Université Blaise Pascal, CNRS, 24 av. des Landais, BP 80026, Aubière Cedex 63171, France; E-Mails: n.montoux@opgc.univ-bpclermont.fr (N.M.); A.Chauvigne@opgc.univ-bpclermont.fr (A.C.); Maxime.Hervo@meteoswiss.ch (M.H.); K.Sellegri@opgc.univ-bpclermont.fr (K.S.); 3 Institut Pascal—UMR 6602, Université Blaise Pascal, CNRS, 24 av. des Landais, BP 80026, Aubière Cedex 63171, France; E-Mail: francois.reveret@lasmea.univ-bpclermont.fr; 4 Federal Office of Meteorology and Climatology, MeteoSwiss, Payerne 1530, Switzerland; 5 Istituto di Scienze dell'Atmosfera e del Clima, Consiglio Nazionale Delle Ricerche, Roma 00133, Italy; E-Mail: d.dionisi@isac.cnr.it; 6 Observatoire de Sciences de l'Univers-Réunion, UMS 3365, CNRS, Université de la Réunion, Saint Denis de la Réunion 97744, France; E-Mail: guillaume.payen@univ-reunion.fr

**Keywords:** atmospheric remote sensing measurements, LIDAR, automation, water vapour, aerosols and cirrus vertical profiles

## Abstract

We present a Rayleigh-Mie-Raman LIDAR system in operation at Clermont-Ferrand (France) since 2008. The system provides continuous vertical tropospheric profiles of aerosols, cirrus optical properties and water vapour mixing ratio. Located in proximity to the high altitude Puy de Dôme station, labelled as the GAW global station PUY since August 2014, it is a useful tool to describe the boundary layer dynamics and hence interpret *in situ* measurements. This LIDAR has been upgraded with specific hardware/software developments and laboratory calibrations in order to improve the quality of the profiles, calibrate the depolarization ratio, and increase the automation of operation. As a result, we provide a climatological water vapour profile analysis for the 2009–2013 period, showing an annual cycle with a winter minimum and a summer maximum, consistent with in-situ observations at the PUY station. An overview of a preliminary climatology of cirrus clouds frequency shows that in 2014, more than 30% of days present cirrus events. Finally, the backscatter coefficient profile observed on 27 September 2014 shows the capacity of the system to detect cirrus clouds at 13 km altitude, in presence of aerosols below the 5 km altitude.

## 1. Introduction

Since the beginning of the 20th century, the increase of anthropogenic atmospheric emissions has induced an evolution of the atmospheric composition which needs to be surveyed and understood, in order to improve climate projections [1]. The satellites provide observations at a global scale, but with a low spatial resolution. They may be completed by *in situ* and remote sensing observations from ground based stations in order to allow the study of dynamical and physico-chemical processes in all their complexity. To fulfil this objective, these observations have to be organized in networks. At a worldwide level, some measurements stations are selected to contribute to the Global Atmospheric Watch (GAW) program of the World Meteorological Organization (WMO). These stations provide reliable data on the physical properties and chemical composition of the atmosphere and contribute to the survey of climate. At the European level, infrastructures such as the Aerosols, Clouds, and Trace gases Research InfraStructure (ACTRIS) network and its component European Aerosol Research LIDAR Network (EARLINET) [2]), or the GCOS Reference Upper Air Network (GRUAN) [3] allowed researchers to homogenize long time series of observations and to implement standard operating procedures of measurements. At the French level, observations are organized around actions of the National Centre for Scientific Research (CNRS), Universities and other ministerial/regional structures. Recent initiatives have the same objective for research and measurements of atmospheric water (ROSEA), aerosols (ORAURE) and gases (GREAT GASES).

In addition to the data quality control, instrumental monitoring, measurement and data processing procedures, it is important to develop a synergy between *in situ* and remote sensing measurements, in order to comprehend atmospheric processes in their entirety and complexity. The atmospheric site PUY is labelled as a GAW global station since August 2014 (and was a GAW regional station before), located around Clermont-Ferrand, a city of 150,000 inhabitants, near the centre of France, at the foothills of the “Chaîne des Puys” mountain ridge. It hosts *in-situ* and remote sensing instruments, deployed in three sites at different altitudes, distant from less than 15 km far away from each other. The Puy de Dôme station (1465 m above sea level) is more than 50% of the time in clouds [4]. Hence, it is dedicated to cloud and aerosol *in-situ* observations [5]. A second site located at Opme (680 m) hosts a VHF radar used to document the dynamics and the origin of the air masses. A third site located at the university campus at Clermont-Ferrand (410 m) hosts some passive remote sensing instruments, X band and micro rain radars [6] and a LIDAR system since 2008. This LIDAR is dedicated to the observation of aerosols, cirrus clouds and water vapour and takes part of the GAW Aerosol LIDAR Observation Network (GALION), ACTRIS, ORAURE and ROSEA networks. It has been used to provide, combined with *in situ* measurements, optical and physical characterization of the volcanic ash of the Eyjafjallajökull eruption in April–May 2010 [7]. The objective of this paper is to provide a detailed description of the LIDAR system, the scientific and technical motivations for improvements and the modifications made on the system. Then, description of the data processing, examples of atmospheric profiles and overview of the database are provided.

## 2. Description of the LIDAR

### 2.1. Principle of the LIDAR

The Light Detection and Ranging (LIDAR) is an active remote sensing instrument developed for the first time in the sixties. Its first applications were in meteorology, where it was used to measure cloud heights [8]. This instrument consists in a laser emitting pulses of light at a given wavelength vertically into the atmosphere and a telescope collecting the light backscattered by the air molecules and particles of the atmosphere. The time elapsed between the emission of a pulse and the reception of the backscattered light allows the calculation of the altitude of the particles and molecules that backscattered the light. Two types of atmospheric scattering can occur: elastic scattering with no wavelength change and inelastic scattering with wavelength modification. For the elastic scattering, when the particles are much smaller than the wavelength of the light, typically for molecules, the Rayleigh scattering theory applies. On the other hand, when the particles have the same size as the wavelength of the light, typically for aerosols and cloud particles, the Mie scattering theory applies for spherical particles. For inelastic scattering, also called Raman scattering, the wavelength shift is characteristic of each molecule and depends mainly of its vibrational-rotational energy. The Raman scattering is many order of magnitude lower than the elastic scattering [9]. The light collected by the telescope must be separated according to the wavelengths by optical devices and then recorded. After processing, depending on the wavelengths emitted and the wavelengths detected, the LIDAR can allow to retrieve wind, temperature, chemical composition (water vapour, ozone, …) and particles properties profiles (aerosol and cirrus cloud extinction).

### 2.2. Description of the Original System

The CO-PDD LIDAR is a Rayleigh-Mie and Raman LIDAR dedicated to aerosols, cirrus and water vapour measurements. It was designed by Gordien Strato and built by Raymetrics [10] in 2007. It uses a Quantel CFR-400 [11] laser to emit pulses of about 7 ns at 355 nm. The energy per pulse is about 60 mJ, with a repetition rate of 10 Hz. Thanks to a Galilean telescope that expands ten times the beam diameter (50 mm), the divergence of the laser beam is 0.14 mrad. Only the third harmonic (355 nm) is transmitted in the atmosphere for eye safety reason. The fundamental (1064 nm) and second (532 nm) harmonics are filtered with two beam splitters that are also used for the laser alignment with the receiving telescope. The emitted beam is polarized linearly.

**Figure 1.**
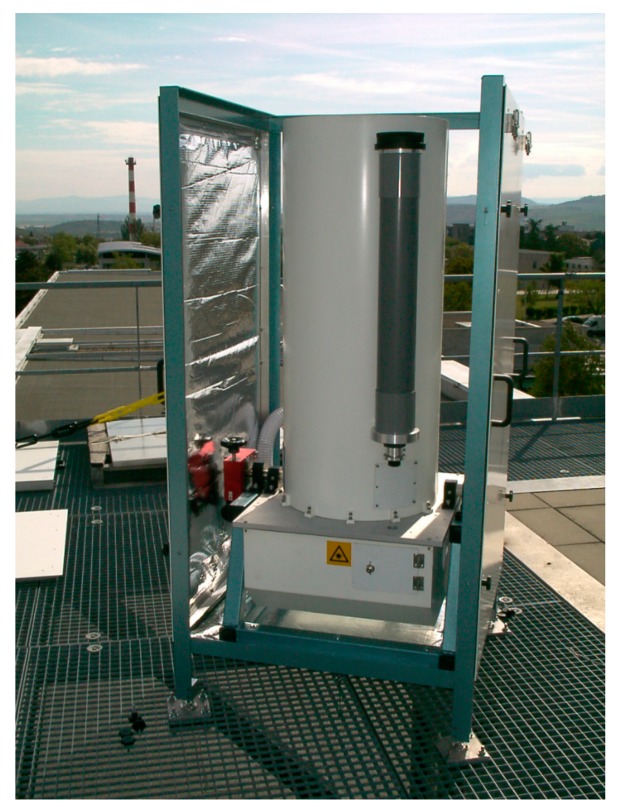
Optical part of the LIDAR on the roof of Clermont-Ferrand University.

The receiving telescope is a 400 mm Cassegrain telescope with a 4 m focal length. A field stops set from 1 mm to 4 mm permits to change the telescope field of view from 0.25 to 1 mrad. The biggest field of view is used for lowest troposphere measurements while a smaller field of view is used for cirrus measurement for instance. A lens located 150 mm behind the focus of the telescope collimates the light at the entrance of the receiving box. The optical part of the LIDAR is deployed outside, on the roof, allowing the opportunity to tilt the LIDAR in the Puy de Dôme direction (Figure 1).

The receiving box is dedicated to the splitting of the receiving laser light in 4 different channels:

- 1 elastic channel with the same polarization as the laser one, called p
- 1 elastic channel with a cross polarization, called s
- 1 inelastic channel for nitrogen Raman scattering at 387 nm
- 1 inelastic channel for water vapour Raman scattering at 408 nm.

**Figure 2.**
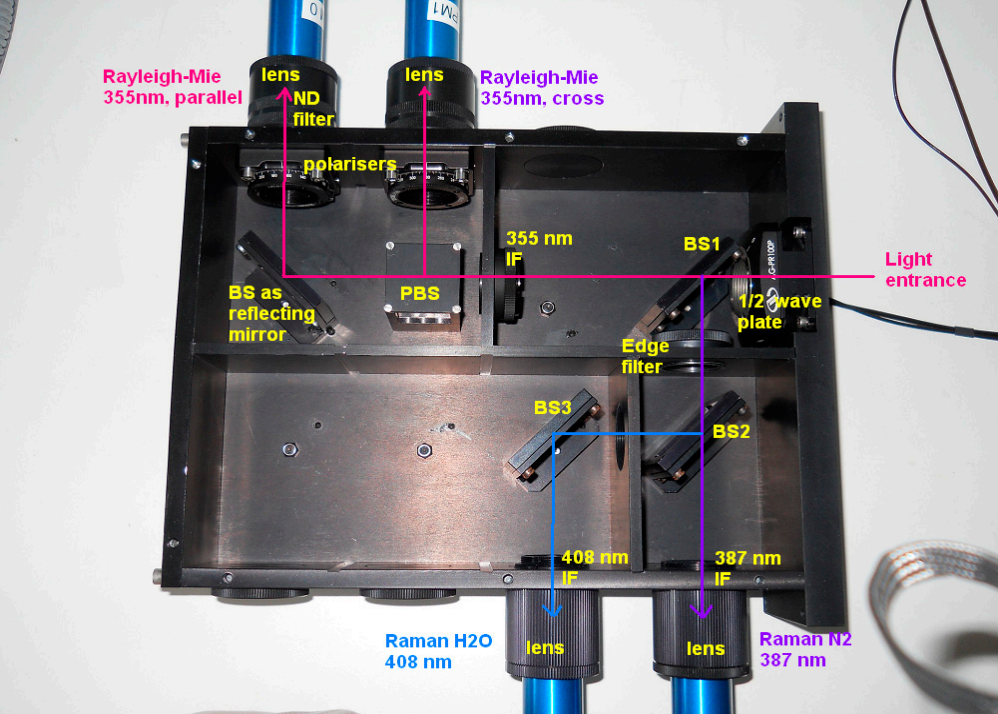
Inside of the receiving box. The input light comes from the right side. ND means neutral density, BS: beam splitter, PBS: polarization beam splitter cube and IF: interference filter.

Figure 2 shows the current receiving box of the LIDAR. After passing through a half-wave plate, the 355 nm is transmitted to the left and the longer wavelengths are reflected downward by a first beam splitter. The parallel and cross polarization are separated by a polarization beam splitter cube, while the Raman channels are separated with a second dichroïc beam splitter. The features of this box will be explained in more details in the Sections 4.1 and 4.2.

Four Licel photomultiplier modules based on R7400 Hamamatsu's PMT tubes receive the optical signal from each channel. Those signals are transmitted to a Licel transient recorder with 7 m long shielded 50 Ω Lemo cables. This electronical part of the LIDAR is installed into an air conditioned room on the floor below the optical part of the LIDAR.

For each elastic channel, the Licel transient recorder allows acquiring simultaneously in analogue and photo-counting mode. For each inelastic channel, the transient recorder can only acquire in photo-counting mode. The acquisition software is based on a Raymetrics labview program. We implemented some improvements on this software in order to:

- manage automatically the measurements (Section 5.1)
- display and publish real-time quick looks (Section 5.2)
- manage scanning capability (Section 3.2).

The raw vertical resolution of the output data is 7.5 m and the raw minimum time resolution is 10 s, corresponding to the accumulation of signals from 100 laser shots. Depending on the parameter studied (aerosols, cirrus clouds or water vapour), the vertical and temporal resolution can be degraded to increase the signal to noise ratio. Except for measurements dedicated to the study of the cirrus clouds heterogeneities where the raw data resolution is kept, data are usually acquired at 1-min time resolution (600 shots). Due to the biaxial configuration of the LIDAR system (the telescope optical axis and the laser beam axis are separated from 300 mm), the laser beam overlaps completely the field of view of the telescope at a distance measured about 1000 m above the LIDAR, with the 4 mm field stop. An overlap correction must be applied to process the measurement data when the overlap is not full. Many methods exist to estimate this overlap function as ray tracing calculation or horizontal LIDAR measurements assuming that the atmosphere is homogeneous and that the alignment of the system remains the same horizontally and vertically (no deformation of the telescope).

**Figure 3.**
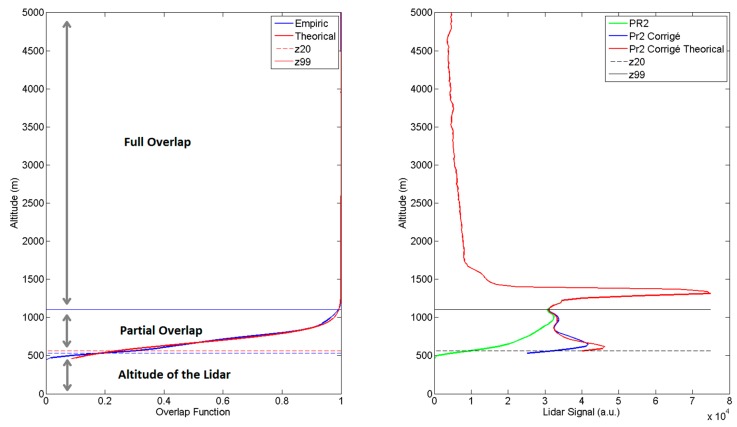
(**Left)**: Measured (blue) and theoretical (red) overlap function; (**Right)**: range and background corrected LIDAR signal (green). Same signal corrected with measured (blue) and with theoretical overlap function (red). The dash line represents the altitude where less than 20% of the signal is measured (z20). The continuous line represents the altitude where more than 99% of the signal is measured (z99).

Figure 3 presents the overlap function calculated theoretically [12] and estimated with horizontal measurements (with the 4 mm field stop). The theoretical and the measured function are in agreement. Thus it seems that the telescope is not deformed when the LIDAR is measuring horizontally.

In the next section the efforts made in order to improve automatic measurements capabilities will be presented.

## 3. Hardware Developments

### 3.1. Thermal and Protections Improvements

The optical part of the LIDAR has been upgraded with a window in order to protect it against precipitations and to allow operation in any weather conditions. The glass used for the window must transmit in UVA range and must be an amorphous material, in order to not modify the polarization properties of the emitted and received light. In addition, there is a cost issue due to the quite large dimension of the optical component (850 × 850 mm) and because it is an annex component, not essential to carry out a LIDAR measurement. A borosilicate glass named BOROFLOAT^®^33 and manufactured by the Schott Company met these criteria. The transmittance in the UV range is about 90%. No anti-reflection (AR) coating is present on the glass for economical reason (an AR coating would have been necessary for the 3 wavelengths received by the telescope: 355 nm, 387 nm and 408 nm). Consequently, a loss of 8% can be added due to the reflections on the both surfaces. Despite of these losses, the global transmission remains acceptable regarding the strength of the signals received by the PMTs. This window is glued on a 900 mm square aluminium frame that covers the enclosure of the LIDAR. This cover is also a bit tilted (about 1°) to avoid light return into the laser.

**Figure 4.**
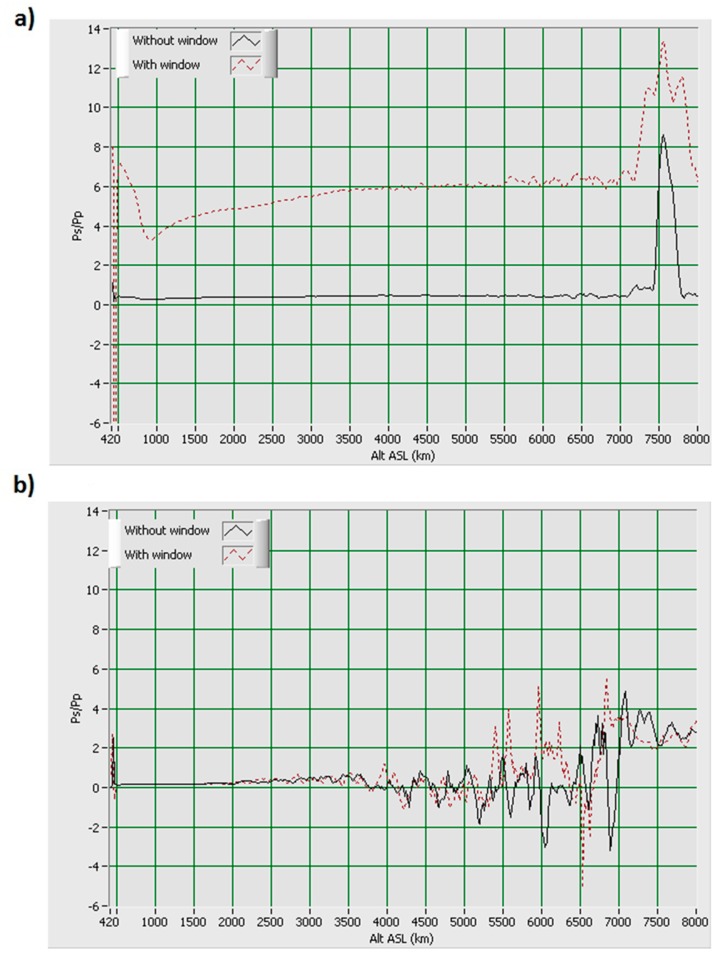
(**a**) Ratio between the s and p channels, with (dotted red) and without (black) the window when it was glued with an inappropriate glue; (**b**) Ratio between the s and p channels, with (dotted red) and without (black) window in the new cover design with silicon glue.

A first issue is the induced depolarization due to mechanical stressed applied on the glass. We noticed that the sag of the window does not affect the polarization of the light. On the contrary the stress of the aluminium frame on the glass (due to the thermal dilatation) has to be taken into account. The coefficient of linear thermal expansion (CTE) of the Borofloat^®^33 is 3.25 × 10^−6^ K^−1^ while it is 24 × 10^−6^ K^−1^ for aluminium. For instance, a decrease of 20 °C on a 900 mm square will contract the aluminium frame by 0.432 mm while the glass will be contracted by 0.0585 mm. If the glass is fixed on the frame with a polyurethane glue, the frame will apply on the glass a sufficient stress to induce birefringence in this material, and then a depolarization of the light depending on the temperature, which cannot be acceptable for the LIDAR depolarization ratio measurement. It means that the glass must not be stressed by the frame, and that can be obtained by using a silicon glue which remains elastic even during long period. The Figure 4 shows how much inappropriate glue can influence the ratio between the parallel (p) and cross (s) channels. On the contrary one cannot detect any significant bias between p and s channels when using elastic glue or a mounting without any stress on the window. The agreement between the 2 curves of the Figure 4b is indeed quite good, the small differences can be explained by the atmospheric variability during the delay of about 3 min between the two measurements.

A second issue, not really depending on the window, is the solar radiation hitting the inner of the telescope tube during spring and summertime. Such radiations induce a differential heating between the top and the bottom of the tube, and then a misalignment of the telescope. This misalignment can be so severe that the LIDAR signal can be completely lost for all channels. Thus it is also necessary to protect the telescope with a sun shield. This sun shield consists of a cylinder of 600 mm high with a 500 mm diameter. When the LIDAR is oriented to the zenith, the axe of this tube corresponds to the telescope one. A hole that fits with the laser beam axes allows the laser to be emitted in the atmosphere. The cover was manufactured with polypropylene in order not to disturb the X-band radar located 4 m from the LIDAR. It has also been closed with two Borofloat^®^33 windows: one for the telescope and the other for the laser beam. Unfortunately the CTE of the polypropylene is 150 × 10^−6^ K^−1^ implying that the expansion of the tube diameter should be 1.5 mm for a 20 °C variation. As a result the expansion is bigger than the expansion of the cover with the aluminium frame. As a precaution, this circular window was not glued like the others, and was solely lied on a gasket and fixed with 4 iron knees. Thus, the cover can expand without stressing the window.

Thanks to this protecting cover, the optics of the LIDAR can then be thermoregulated. Instead of installing an air conditioner on the enclosure, we decided to take advantage of the air conditioned room where the electronic part is installed, so a fan was fixed in the room just above the cooling unit in order to insufflate the air of the room inside the optical part enclosure, through an insulated hose.

Two thermostats in the enclosure allow stopping the fan when regulation is not necessary. This thermal regulation has several advantages:

- This system is not expensive and its implementation is quite easy.
- There is no electromagnetic disturbance on the signal due to an added cooling unit.
- The enclosure is a bit pressurized, and the incoming air flux is filtered. Thus the optical part is preserved from outside dust.
- It is a reverse air conditioning since the air flux is cooler in summer and warmer in winter, than the outside air (during winter the air conditioner of the room is stopped, so the LIDAR enclosure takes advantage of the room heating).
- The air flux is powerful enough to defrost the window.
- It provides better stability conditions for the laser power.

### 3.2. Scanning System

One interesting feature of the LIDAR was the possibility to incline it until it reaches the top of the Puy de Dôme volcano, where the PUY atmospheric station is located. At the origin, the LIDAR could be manually tilted thanks to a manual reduction gear. Due to a relative inaccuracy of this method regarding the tilt angle, we implemented a motorized scanning system based on an 8.5 Nm stepper motor, and that uses the reduction gear. The multiplication factor of the gear is 60 and the motor is set to give a 0.06° resolution per step. A photo-sensor was mounted on the frame of the moving parts of the LIDAR in order to give the home position, corresponding to the zenithal direction, a software command allows to move the LIDAR to this home position. The reproducibility of the angle positions was tested by tilting the LIDAR down to the PDD direction. When the laser beam touches the horizon, one can see a peak appearing at about 11.8 km due to the backscattering of the soil of the mountain. So this signal always appears at the same angle with an accuracy of 0.1°. Despite the opportunities provided by this system, scanning measurements can have some drawbacks:

- Scanning measurements needs to open the enclosure of the optical LIDAR part, which means that the LIDAR is not protected any more against precipitations and dust. It cannot be thermo-regulated as well.
- The alignment of the LIDAR could be sensitive to the telescope angle when tilted, so the overlap function could change.
- Scanning can take much more time than single vertical profiles. So it may not be adapted for the measurement of a fast event (e.g., quick variations of the boundary layer height).

The software for scanning management was integrated into the Raymetrics acquisition code, in which the user can choose the starting and ending times, the step angle, and the direction of the scan (*i.e.*, upward or downward). Between each scan direction, the acquisition is stopped in order to get one measurement file per angle measurement. The angle is also recorded automatically in the header of the file. The principle of the acquisition software will be described in more details in Section 5.

## 4. Optical Developments

### 4.1. Depolarisation Calibration Device

The original LIDAR had the capability to characterize the depolarizing properties of particles from the combination of the p and s elastic channels. Calibration of the depolarization ratio requires to the knowledge of the depolarization ratio in a range free of aerosols. However, the presence of very depolarizing particles, even at very low concentrations, can induce a significant bias compared to the assumed molecular depolarization ratio [13]. For this reason we implemented the depolarization calibration device based on a −45°/+45° calibration procedure. A half-wave plate mounted in a motorized rotative stage allows a very high angular accuracy. This half-wave plate is placed in front of all optical components that can have some diattenuation, and particularly the first dichroic beam splitter as shown in the Figure 2. Two polariser plates were added as well in front of the p and s PMTs in order to get rid of the cross-talk, as explained in Section 4.2.

By rotating the half-wave plate, it is possible to correct the laser polarization axe offset according to the receiving optics polarization axe [14]. Hence calibration measurements can be carried out by rotating the half-wave plate from this position to ±22.5° (*i.e.*, a rotation of ±45° for polarisation). Considering that:

(1) $$\delta^{*}\left(\theta \right)=\frac{{\text{P}}_{\text{R}}(\theta )}{{\text{P}}_{\text{T}}(\theta )}$$

where PR(θ) and PT(θ) are the received signals on the reflected and transmitted channels respectively by the PBS cube (in this case the transmitted is the parallel polarization and the reflected is the cross one), when the polarization axe is tilted at an angle θ by the half-wave plate. Then the volume depolarisation ratio is:

(2) $$\delta^{\nu }=\frac{\delta^{*}(0{}^{\circ})}{\sqrt{\delta^{*}\left(+45{}^{\circ}\right)\cdot \delta^{*}(-45{}^{\circ})}}$$

If the transmission properties of the optics of the receiving box are known, depending on the polarization, it is possible to get the relative amplification factor V*

(3) $${\text{V}}^{*}=\frac{{\text{T}}_{\text{P}}}{{\text{R}}_{\text{S}}}\cdot \sqrt{\delta^{*}\left(+45{}^{\circ}\right)\cdot \delta^{*}(-45{}^{\circ})}$$

where T_P_ and R_S_ are respectively the global transmission coefficient of the parallel signal, and the global “reflectance” of the cross signal (“reflectance” is incorrect since only the PBS cube reflects this signal in fact). The method used to get those results is detailed in the appendix. The T_P_/R_S_ ratios can be determined by swapping the p and s photomultipliers while the polarisation is rotated by 90° with the half-wave plate, which swaps the p and s signal:

(4) $$\frac{{\text{T}}_{\text{P}}}{{\text{R}}_{\text{S}}}=\frac{{\text{P}}_{\text{T}}(90{}^{\circ})}{{\text{P}}_{\text{R}}(0{}^{\circ})}=\frac{{\text{P}}_{\text{T}}(0{}^{\circ})}{{\text{P}}_{\text{R}}(90{}^{\circ})}$$

Another way to calibrate the depolarization ratio is to characterize the transmission of the whole receiving box for each channel, which has been achieved with the collaboration of the Institut Pascal at Clermont-Ferrand.

### 4.2. Optical Box Characterization

In order to calibrate the depolarization ratio, it is necessary to know the transmission on the parallel and perpendicular channels of the optical box. It is also important to check the quality of each device from the optical box to understand the limits of capability of this LIDAR system. For this purpose, we used optical spectroscopy measurements available at the Institut Pascal. In this setup, a xenon lamp with a polarizer is used for the excitation. This configuration allows transmission measurements with linear parallel or perpendicular polarized light. Then the light is focused on the slit of a 1 m focal length monochromator (FHR 1000), used to separate spatially the incident wavelengths, and an UV-enhanced CCD camera records the signal.

**Figure 5.**
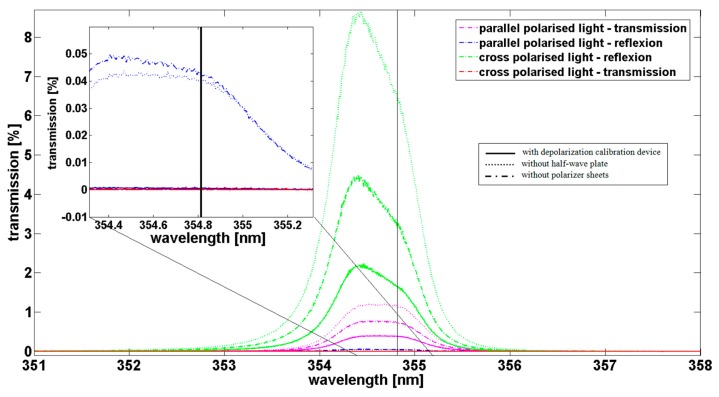
Transmission of the whole optical box in different configurations for the Rayleigh channels: Solid lines: half-wave plate and polarizer plates presents, dotted lines: without polarizer plates and half-wave plate, dashed lines: without polarizer plates but with half-wave plate present.

Before July 2013, *i.e.*, before the implementation of the calibration's device, the transmission at 354.8 nm was of about 1.16% on the parallel channel (in dotted purple line on Figure 5) and 6.37% on the perpendicular channel (in dotted green line on Figure 5) without taking into account the presence of identical coloured filters in front of the photomultipliers. With the attenuation of the coloured filters, the transmission at 354.8 nm was of about 0.93% on the parallel channel and 5.16% on the perpendicular channel. Thus the total backscatter signal P due to the presence of molecules and particles in the atmosphere, could be calculated using the Equation (5):

(5) $$\text{P}\;=\;{\text{P}}_{\text{T}}+f{\text{P}}_{\text{R}}\;\text{with}\;f=\frac{0.93}{5.16}=0.181$$

where P_T_ and P_R_ are the received signals on the parallel polarization (index T for transmitted) and cross polarization (index R for reflected) channels respectively and *f* is the coefficient taking into account the differential optical attenuation between both channels. However the signals received on the two photomultipliers are not completely pure in terms of polarization. On the parallel channel, less than 0.02% of the (parallel and cross) light received is cross-polarized (in dotted red line in the Figure 5) while on the cross-polarized channel, about 0.63% of the light received is parallel polarized (in dotted blue line in the Figure 5). This phenomenon called “cross-talk” means that the beam splitter cube is not perfect and a small amount of parallel polarized light is reflected on the photomultipliers instead of being transmitted. As in the atmosphere the parallel polarized light signal is most of the time stronger than the cross polarized light signal (about two orders of magnitude for molecular backscattering), this “cross-talk” must be avoided to deduce a reliable volume depolarization. To avoid this cross-talk, a polariser plate has been added in front of each photomultiplier to remove the stray light on 3 April 2013. On the Figure 5, the signals are greater without the polariser plates (dashed lines) than with the polariser plates (solid lines) that is expected because those polariser plates attenuate quite a lot of light at 355 nm. Thus, those polariser plates replace the coloured filter in order to keep an equivalent attenuation. With these polariser plates, the cross-talk decreases from 0.63% to 0.03% on the cross-polarized channel (respectively dashed and solid blue lines). The half-wave plate located in front of the whole optical box insures a good alignment of the beam splitter cube with the laser polarization plane. The half-wave plate induces a small added attenuation (transmission lower for the dashed lines than the dotted lines) because it is an uncoated component, in order to transmit the three useful wavelengths (*i.e.*, 355 nm, 387 nm and 408 nm). To summarize, the factor *f* is 0.181 (or 0.179 with cross-talk taken into account) before the calibration of the depolarization (with the neutral density filter in front of the parallel channel taken into account). The factor *f* becomes 0.229 (or 0.226 with cross-talk taken into account) without the polariser plates but with the half-wave plate and 0.229 (or 0.228 with cross-talk taking into account) with the polariser plates and the half-wave plate.

**Figure 6.**
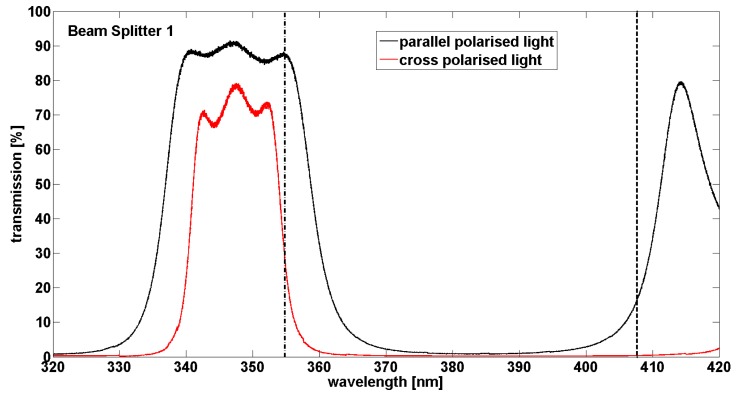
Transmission of the beam splitter 1 according to the wavelength.

One important point was underlined by these tests. As shown on the Figure 5, the transmission of the optical box depends strongly on the wavelength. A variation of ±0.1 nm (from 354.7 nm to 354.9 nm) induces a change in the transmission for the parallel channel from 0.39% down to 0.34% which represents a relative variation in transmission of 14% for the configuration with the polariser plates and the half-wave plate for example. Likewise, for the perpendicular channel, the transmission varies from 1.84% down to 1.41% which represents a relative variation of 26%. This has a strong impact on the factor *f* which can vary from 0.214 up to 0.241 that represents a relative uncertainty of 12% not acceptable for the calculation of the volume depolarization ratio. As temperature variation in the system can induce such kind of shift in wavelength, it is crucial to understand the origin of this variation. By studying the transmission of each optical device of the box individually, the beam splitter 1 shown on the Figure 2 is involved. This beam splitter transmits the elastic signals at 355 nm and reflects the Raman signals. At 354.8 ± 0.1 nm the transmission of the parallel component is 87.2% ± 0.6% while the transmission of the perpendicular component is 27.9% ± 2.3% (Figure 6). As in the atmosphere, the perpendicular component is quite weak, it is crucial to have a strong transmission for this component with no dependence in wavelength (*i.e.*, temperature). In addition, the beam splitter transmits about 17% of the parallel component at 408 nm which is the water vapour signal (Figure 6). This signal is thus not transmitted on the Raman 408 nm photomultiplier. As the 408 nm signal is quite weak in the atmosphere, this feature reduces again the signal to noise ratio and prevent measurements above 10 km. To resolve these problems, a new design of the optical box is planned in the future with the change of the actual beam splitter for a low pass beam splitter and the swap between elastic and inelastic channels.

**Figure 7.**
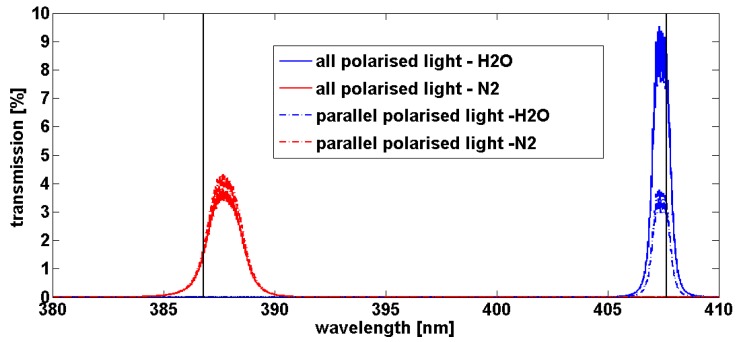
Transmission of the whole optical box for the Raman channels: nitrogen in red and water vapour in blue.

Concerning the Raman channels, the Edge filter in Figure 2 has a transmission of about 79% for the nitrogen and 85% for the water vapour. The global transmission on the Raman channels is shown on Figure 7. For nitrogen, the maximum transmission is around 387.7 nm with about 4.3% and for water vapour, the maximum transmission is around 407.3 nm with 3.8% for parallel polarized light. The full width at half maximum is 1.7 nm for nitrogen and 0.8 nm for water vapour. Thus the transmissions at the wavelengths due to the vibrational state transition of the molecules (386.8 nm for N_2_ and 407.663 nm for H_2_O) are quite low: 1.7% for nitrogen and about 2.9% for water vapour. Even if the transmission windows seem not well centred on 386.8 nm for N_2_ and 407.663 nm for H_2_O, this could be due to a misalignment of the optical box during the tests. More precisely, it could be due to a misalignment of the beam splitters 2 and 3 in the optical box or to a small variation of the 45° incidence angle on the beam splitter 1 because of experimental difficulties to measure transmission of a set of optics. However, it underlines the requirement of a very accurate alignment of all the devices in the optical box. For this, the fixation of the beam splitters with two screws could be not sufficient. Another point to notice is a weak transmission observed in the purple (maximum of 0.044% at 413 nm) on the water vapour channel and in the blue (maximum of 0.052% at 448.9 nm) for the nitrogen channel (not shown). Even if these transmissions are respectively more than 64% and 33% weaker than the transmission at 407.663 nm (for H_2_O) and 386.8 nm (for N_2_), the Raman signals in the atmosphere are very weak and can be polluted by the solar radiation during the day (whose maximum is in the blue) and to a lesser extent, the moon radiation (whose maximum is in the red) during the night. This feature added to the solar light received in the Raman lines could explain the noise observed during the daytime Raman measurements which prevent retrieving water vapour content.

## 5. Software Developments

### 5.1. The Automatic Measurement Management Software

The principle of the Licel/Raymetrics labview acquisition software is based on a state-machine with events management. We implemented a complementary module for the automatic and for the scanning management.

**Figure 8.**
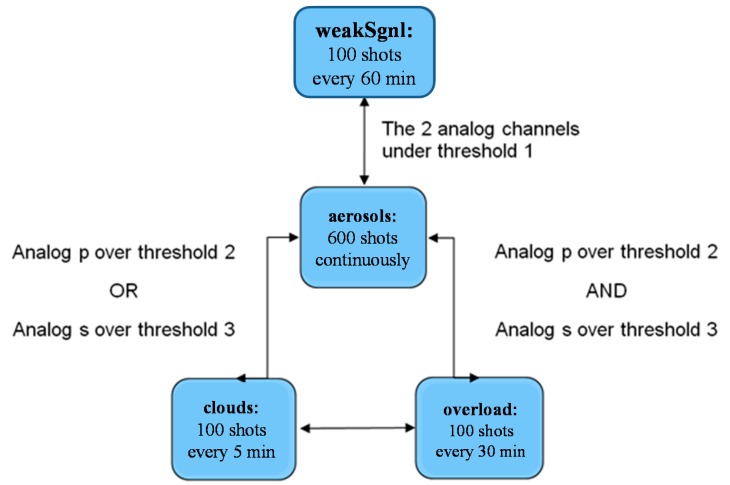
Management of the measurement conditions.

For the aerosols and water vapour measurements, a file with the different channels profiles is recorded each minute. Instead of entering in a state where a new acquisition restarts, the program enters in a state where the behaviour of the signals is analysed in order to determine the next state (e.g., suspending or continuing the measurements). The maximum peaks of both parallel and cross analogue channels are compared with three different thresholds. The first one is used to check if the backscattered signals are powerful enough. The second and the third are used to check if the parallel and perpendicular (respectively) signals are too powerful. Figure 8 shows the scheme of the actions the program will carry out depending on the comparison of the analogue signals with the thresholds. Here is a description of each case:

- Weak signal: this situation could occur when some frost or condensation occults the signal;
- Aerosols: normal conditions for continuous measurements (not only for aerosols but for water vapour as well);
- Clouds: in that case, low clouds occultation makes aerosols and water vapour processing impossible;
- Overload: one (or even both) signal is too strong, so it could damage a PMT.

When the program suspends the measurements, it actually stops the laser firing, so the acquisition, which is triggered by the laser, is suspended as well. A string flag is written in the header of the files to discriminate usable profiles (e.g., “aerosols”, “clouds”, “overload”). Such an algorithm to suspend the measurements is quite simple, but it is sufficient for the low clouds files segregation. However the efficiency becomes much weaker in case of rain, due to the fact that raindrops do not backscatter the signal as strongly as cloud particles. Moreover, the droplets on the protective window can also attenuate the emitted and the received signal. It means that during a rain event, the thresholds do not match any more the management schedule and the LIDAR will keep on acquiring measurements while they should be suspended. One could overcome this issue by adding a more advanced processing that could, for instance, analyse the exponential decreasing shape of the signal beyond a detected peak signal. An analysis of the signal to noise ratio could be another method. Whatever the chosen means, such a processing should not be applied in the frame of the acquisition state-machine, because it could delay the start of the next acquisition profile. So this processing must be done in parallel to the acquisition. The principle will be described in the Section 5.2.

The last point regarding the automatic measurement management is the capability to start and stop automatically the Raman channels depending on day or night time. The 355 nm cross channel is the most daylight sensitive elastic channel in photo-counting mode, because the cross signal is not so attenuated as the parallel one in front of the PMT. So the background noise variations are strong enough to set a threshold between day and night. Then, inside the same state as for low clouds conditions management, the program switches on the power supplying of the Raman PMTs and the Raman channels recording when the background noise of the 355 nm photo-counting cross channel becomes below this threshold at sun set. The program carries out the contrary at sunrise.

### 5.2. Display and Publication of Temporal Serials in Real Time

The acquisition state-machine should carry out a minimum of processing tasks in order not to slow down the acquisition recording speed. However, it is always possible to use the acquisition software in parallel for processing, displaying, and publishing data, while the LIDAR computer waits for new recording from the Licel transient recorder. This is done by using a producer-consumer pattern where the producer loop is the acquisition state-machine and the consumer is a synchronized loop that runs in parallel. When a new recorded set is ready (*i.e.*, a new data file), this second loop processes the data, adds a new profile to a daily temporal serial and uploads it to an OPGC website page [15] in real time.

At the present time, a level 1 processing is applied and the volume depolarization ratio and the result of a Wavelet Covariance Transform (WCT) technique for boundary layer visualization are published [16]. A climatology of the boundary layer height determined with the WCT algorithm in described in [17].

### 5.3. Aerosols and Cirrus Properties Retrieval

For aerosols and cirrus measurements, the corrections on the signal were implemented in accordance to the ones achieved by the ACTRIS/EARLINET recommendations. The different steps are (with a focus on the original method to get the dark measurement data):

- Discrimination of usable and unusable files (thanks to the string flag described above)
- Data reduction of the spatial resolution (e.g., from 7.5 m resolution to 15 m)
- Search of the closest dark measurements (DM) files that match the measurements setup (*i.e.*, high voltage of the PMTs and amplitude range for analog modes). A DM is a measurement occulting totally the receiving light. Even without incoming light, the analog channels profiles are not completely flat. So the DM purpose is to suppress the contribution of the electronic in the lidar analog profiles the search of the closest DM is based on a recursive algorithm. The starting point of the search is the dataset of measurement. Then the program looks backward for the first DM file detected (thanks to the header flag). If the analog parallel channel set-up does not match the DM's one, the search restarts forward from the last starting point, and so on. After a matching set-up is found for the parallel channel, the search restarts for the cross channel. It means that the dark measurement data can come from a different file between the p and the s channel, if necessary. The most important thing is to get the DM closest to the measurements. However, this recursive algorithm to find the best DM may be replaced by another one based on a state-machine and oriented object programming.
- Temporal averaging of the dataset
- DM correction on the analog channels
- Dead time correction on the photocounting channels
- Trigger delay correction
- Background noise correction
- Savitsky-Golay smoothing
- Gluing between analog and photocounting
- Range correction by the square of the distance

In the case of aerosols measurements, it is still possible to apply an overlap correction and also to calculate at this point the depolarization ratio δ^v^ as described by Equation (2). Then, by using a radiosounding profile, a Rayleigh fit [18] is carried out to determine the reference altitude for the Fernald-Klett inversion [19] to retrieve the aerosols backscattering coefficient profiles [20]. A Raman algorithm for the extinction retrieval has already been tested (Section 6.4) and is under implementation for routine processing, based on [21].

### 5.4. Water Vapour Retrieval

The water vapour mixing ratio is the ratio of the mass of water vapour to the mass of dry air in a given volume of the atmosphere. In the troposphere, nitrogen is almost in a constant proportion of dry air. Then, the water vapour mixing ratio can be derived from the ratio between the H_2_O and the N_2_ Raman backscattered signals (respectively S_H_2_O_ and S_N_2__), using the following expression [22]:

(6) $$\text{q}\left(\text{z}\right)=\text{C}.\Gamma \left(\text{z}\right)\cdot \frac{{\text{S}}_{{\text{H}}_{2}\text{O}}}{{\text{S}}_{{\text{N}}_{2}}}$$

where C is the calibration coefficient and Γ(z) is the atmospheric differential transmission.

The estimation of the calibration coefficient is a crucial issue which is still pending, particularly in NDACC community [23,24]. Though an absolute calibration of the entire LIDAR system is theoretically possible [25], external water vapour measurement (radiosonde or GPS), or model profiles are commonly used to calculate the calibration coefficient because some instrumental parameters such as laser emission stability, transmission of optical parts, or electronic efficiency of PMTs are difficult to precisely evaluate. Concerning the estimation of the calibration coefficient of the water vapour channel of the Clermont-Ferrand system, the use of the vertical columns obtained from GPS measurements performed at the Puy de Dôme and Cezeaux have been evaluated but the GPS station of Puy de Dôme is distant from 12 km and the GPS station from Cezeaux is collocated with the LIDAR but provides the total column of water vapour over the altitude of the site (410 m) when the bottom limit of LIDAR profiles is around 1500 m (Figure 3). In addition, there is no collocated radiosonde measurement available for this site. Consequently, the calibration procedure has been performed normalizing by water vapour mixing ratio from the ECMWF ERA-Interim reanalysis between 3 and 5 km height.

Another important issue is the choice of integration time for the calculation of water vapour mixing ratio profiles. The system provides files every one minute. However, since Raman returns are weaker than Rayleigh-Mie returns, it is necessary to integrate enough time to obtain a vertical profile covering a significant part of the troposphere. Water vapour having a large variability and being governed by processes leading to a succession of independent situation that should not be averaged, the choice of the integration time is a compromise between the accuracy and vertical range of the profile and the temporal variability of water vapour behaviour. In addition to the calculation of one profile for the whole night, we used the methodology presented in [9] to extract automatically quasi-stationary periods and obtain one or several independent water vapour profiles during each acquisition night. Examples of water vapour profiles will be presented in the Section 6.2.

## 6. Overview of the Dataset, Examples of Measurements and Climatology

### 6.1. Scientific Motivations

Water vapour has long been recognized as one of the most important trace gases in the atmosphere. The measurements of water vapour profiles are important for understanding and forecast of the moisture convection, horizontal transport and stratosphere-troposphere exchanges. Water vapour can also be used as a tracer of cloud formation and rainfall event [26]. In addition, the radiative effects of water vapour are of prime importance for the global climate [27]. Accurate measurements of water vapour in the upper troposphere and lower stratosphere are still difficult to perform from space given its very low concentration, its large variability and vertical decrease and stratification [28].

Upper tropospheric clouds, such as cirrus, have also been identified as one important regulator of the radiance balance of the Earth atmosphere system [29]. The net radiative effect depends on the competition between greenhouse and albedo effects that are linked to the microphysics, the height, the temperature and the water vapour density.

Atmospheric aerosols are produced by many sources and have also a significant impact on human health and climate. Since aerosols are mainly emitted close to the surface, their vertical distribution is a good indicator of the atmospheric boundary layer vertical extension that can be assimilated to the first aerosol mixing layer. The dynamics of the boundary layer extracted from the LIDAR located in Clermont-Ferrand is used to interpret in-situ measurements performed at the PUY station.

Moreover, thanks to its ability to characterize aerosol layers that travel at high altitudes and hence that cannot be easily sampled by *in situ* instrumentation, LIDAR measurements are also a key data for analyzing special aerosol events such as Saharan dusts, volcanic and biomass burning plumes, which influence significantly atmospheric properties [17,30,31].

At last, aerosol measurements from a LIDAR research network also permit to complete aerosol forecasting by forcing modelisation exercise through data assimilation techniques [32].

The CO-PDD LIDAR allowing to estimate and survey on a routine basis water vapour and cirrus clouds vertical distributions, we provide in the following sections an overview of the database, examples of profiles and preliminary climatological distributions obtained since 2008.

### 6.2. Dataset

**Figure 9.**
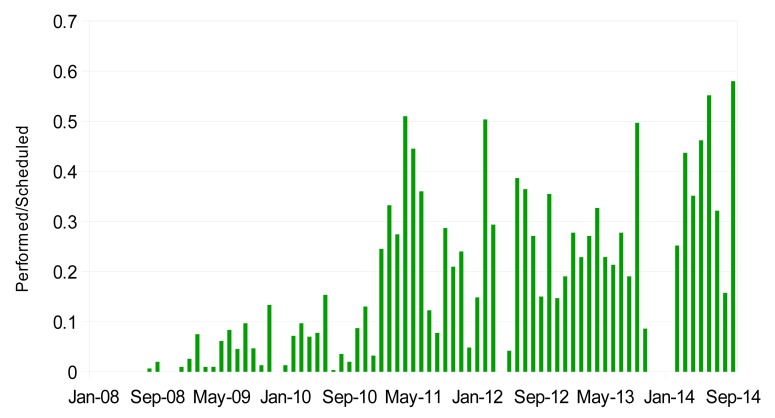
Monthly percentage of time measurement operation of the LIDAR, after removing flagged files as weak signal, low level cloud, tests.

The first tests of Rayleigh-Mie channels were performed in 2008, while Raman channels were tested in 2009. Figure 9 is a histogram showing the monthly percentage of time operation, considering only valid measurement files, *i.e.*, with no test or other kind of files. During the period 2009–2010, due to the manual operation, the system was operating around 10% of the time. The increase to around 30%–50% of the number of measurements per month after 2011 is due to the systematic measurements start. Since 2013 and thanks to the system automation, a better regularity of the measurements is achieved. The number of measurements per month will be limited only by the bad weather conditions. The gap of four months (from October 2013 to January 2014) is due to a maintenance operation of the laser.

**Figure 10.**
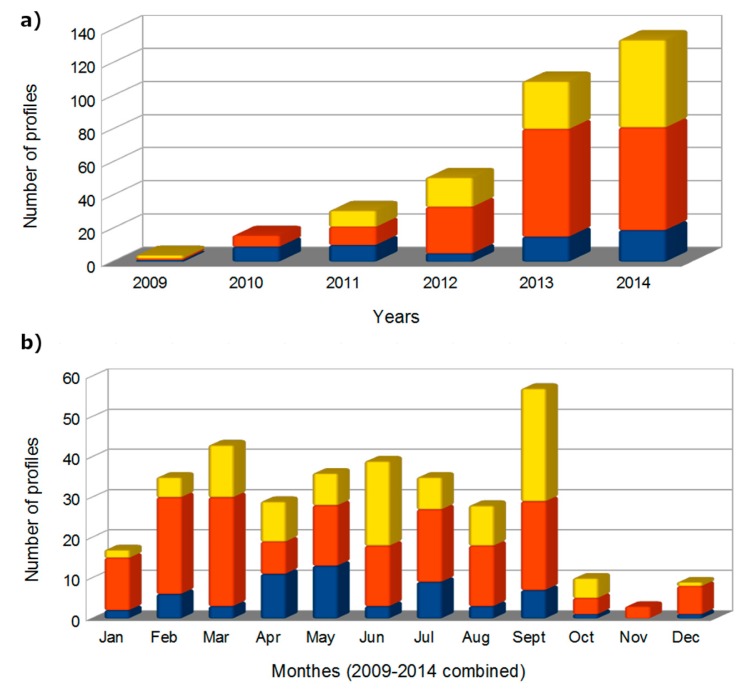
(**a**) Dataset histogram of whole night water vapour LIDAR profiles between June 2009 and September 2014. The vertical upper limit of the profiles are given by colours (blue: <5 km; red: 6–8 km; yellow: >9 km); (**b**) Monthly repartition of profiles.

Regarding more specifically night time Raman water vapour measurements, the number of nights during which a profile reaching at least 4 km was improved year after year, reaching more than 100 night-time profiles in 2013 (Figure 10), and 200 night time profiles during 2009–2013. Based on the methodology regarding the integration time period described in Section 5.4, around 900 independent profiles have been obtained over this period. This database allowed us to establish water vapour climatological profile for this site, presented in the Section 6.2.

**Figure 11.**
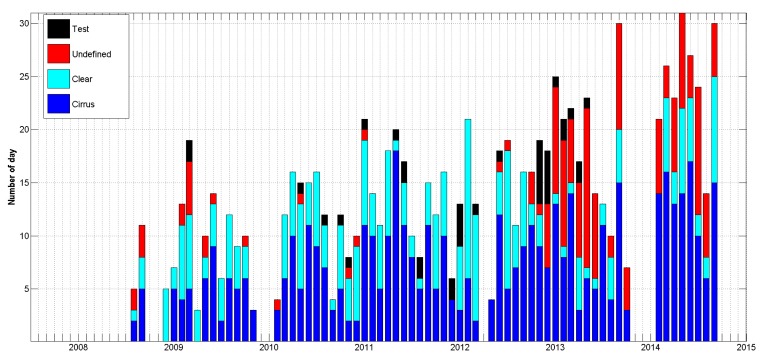
Monthly number of day presenting cirrus signal (blue), clear conditions (cyan), technical test (black) and undefined cases (red) from the 7 years LIDAR dataset.

Since 2008, available LIDAR channels permit to retrieve the depolarization ratio and thus, cirrus covering over Clermont-Ferrand. The selected range in this latitude, in order to classify days into cirrus event, is taken between 5 km and 15 km above ground level. Figure 11 is a histogram showing the monthly number of day presenting cirrus signal, clear condition, technical test or undefined day. Clear conditions are selected from clear sky between 5 km and 15 km and excluding heterogeneous atmosphere under 5 km. Undefined day are most of the time noisy measurements or absence of measurement between 5 km and 15 km due to the presence of thick low clouds (below 5 km) that attenuate the signal. In Figure 11, the number of measurements is again increased since 2013 with the system automation allowing more night time and weekend measurements. We note an increase of undefined days after 2013, because more measurements are performed in low clouds conditions since they are carried out continuously. More and more cirrus signals are retrieved and permit to study cirrus properties with a large dataset. In 2014, more than 30% of days present cirrus events. Even if the presence of cirrus clouds seems to increase with time, a detailed study is needed before drawing such a conclusion. Indeed, the number of measurement hours varies from day to day, depending on the weather conditions (rain, low clouds) and some days classified as cirrus event could have presence of cirrus clouds during all the day as for only few hours.

### 6.3. Water Vapour Measurement Profiles

**Figure 12.**
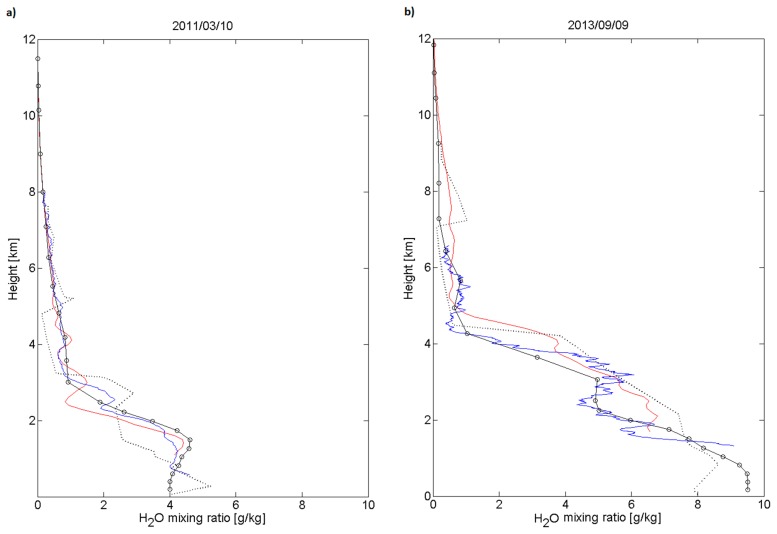
(**a**) Water vapour vertical profiles obtained with LIDAR (blue curve) at Clermont-Ferrand from the 9 March 2011, 18.41 UT to the 10 March 2011, 5.17 UT. The profile is superimposed with a radiosounding profile performed at Nimes (240 km far from the LIDAR) on the 10 March 2011, 0 UT (black dotted line), with a profile obtained from satellite FORMOSAT-3/COSMIC (red curve) on the 10 March 2011, 4 UT at 45.6131°N, 3.6278E, *i.e.*, 43 km far from the LIDAR and with the profile obtained from the ECMWF ERA-Interim reanalysis (black curve one circle by vertical level) on the 10 March 2011, 0 UT, at 45.75°N, 3°E; (**b**) LIDAR profile obtained from the 9 September 2013, 19.15 UT to 22.42 UT; radiosounding profile performed at Nimes on the 10 September 2013, 0 UT; FORMOSAT-3/COSMIC profile on the 9 September 2013, 15 UT at 44.1115°N, 2.8356°E, *i.e.*, 185 km far from the LIDAR and ECMWF ERA-Interim reanalysis on the 10 September 2013, 0 UT, at 45.75°N, 3°E.

Two examples of water vapour LIDAR profiles are given in Figure 12. The profile obtained on the 10 March 2011 reaches 8 km (630 min integration time) and the profile obtained on the 9 September 2013 reaches 6.5 km (57 min integration time). These two profiles have been chosen because a water vapour profile from FORMOSAT-3/COSMIC coincident in time and space are available. FORMOSAT-3/COSMIC (F3C) is a joint Taiwan/US science mission launched in April 2006, for weather, climate, space weather and geodetic research [33]. Biases in COSMIC sounding data are considered to be relatively small and therefore, are expected to be stable in time and space. COSMIC-FORMOSAT 3 water vapour profiles are retrieved using the GPS radio occultation technique, and applying a one-dimensional variational method that makes use of the ECMWF low resolution analysis data as a guess of atmospheric water vapour [34]. The ECMWF ERA-Interim water vapour profiles used for the LIDAR calibration and the radiosounding profiles performed at Nimes (240 km away from Clermont Ferrand) are also presented in Figure 12.

**Figure 13.**
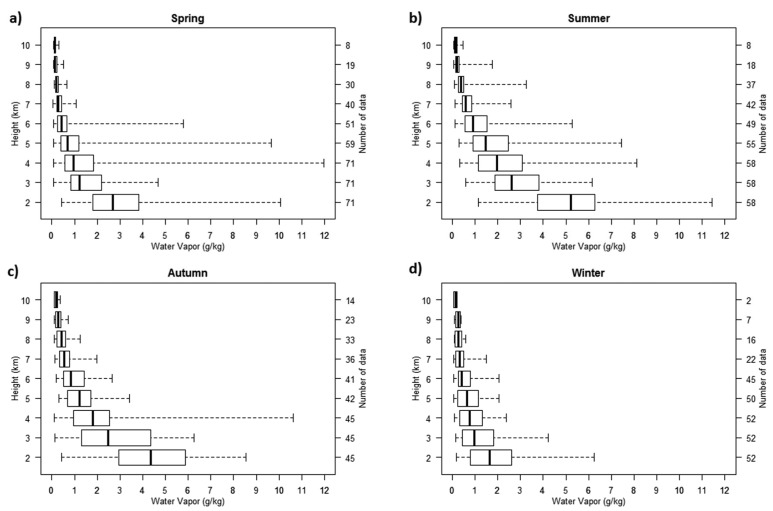
Boxplots of the seasonal profiles of water vapour mixing ratio at Clermont-Ferrand (2009–2013) (**a**) March–April–May; (**b**) June–July–August; (**c**) September–October–November; (**d**) December–January–February.

These two examples display a very good agreement between the four profiles. It has to be noted that around 15 case studies with a spatial and temporal co-location mismatch within 300 km/12 h. Because of the important variability of water vapour in time and space, the agreement is not always as good as in the two examples presented here. The vertical upper limit of the LIDAR profiles can be better than the one obtained on the 10 March 2011 profile, and can reach 11 km. A statistical analysis of spatial and temporal co-location mismatch between FORMOSAT-3/COSMIC and the Italian LIDAR Potenza EArlinet Raman LIDAR (PEARL) in 2008 is presented in [32]. The authors concluded on a good performance of COSMIC in the identification of the vertical gradients of the water vapour field, even though the average difference between the Raman LIDAR and the COSMIC profiles suggests that caution should be taken in using COSMIC data as an absolute or reference measurement of water vapour, in particular in the low and middle troposphere. The climatological seasonal water vapour profiles presented in the Figure 13 show median values at 2 km varying from less than 2 g/kg in winter, to more than 5 g/kg in summer. The water vapour mixing ratio decreases rapidly with height. The variability is lower in winter over the whole troposphere than in the other seasons.

Very few climatologies of water vapour LIDAR profiles on comparable sites have been published, but except local or regional specificities of each site, water vapour distributions observed at Clermont-Ferrand seem in agreement with other measurements previously done at Potenza, Italy [35], Oklahoma, USA [36] California, USA [37], Observatoire de Haute Provence, France [9], or Reunion Island [38].

### 6.4. Aerosols and Cirrus Cloud Optical Properties Example

Aerosols backscattering coefficient profiles are retrieved using Fernald-Klett inversion [19,20]. The inversion is applied on range corrected signal (Pr^2^ signal) using a specific LIDAR ratio (LR). For cirrus clouds, the lidar ratio used is of 20 sr according to the lidar ratios usually measured at 355 nm at mid-latitudes [39,40]. For Boundary Layer and forest fire smoke aerosols, the lidar ratios used are of 58 sr and 46 sr respectively, according the multi air mass study of [41]. In order to separate cirrus and aerosol signals, the boundary layer aerosol LIDAR ratio is applied up to 3 km, the forest fire smoke LIDAR ratio between 3 km and 8 km and the cirrus LIDAR ratio above. A more accurate method to retrieve the LIDAR ratio consist in using Raman LIDAR channel and is currently in development on the Clermont-Ferrand LIDAR system.

**Figure 14.**
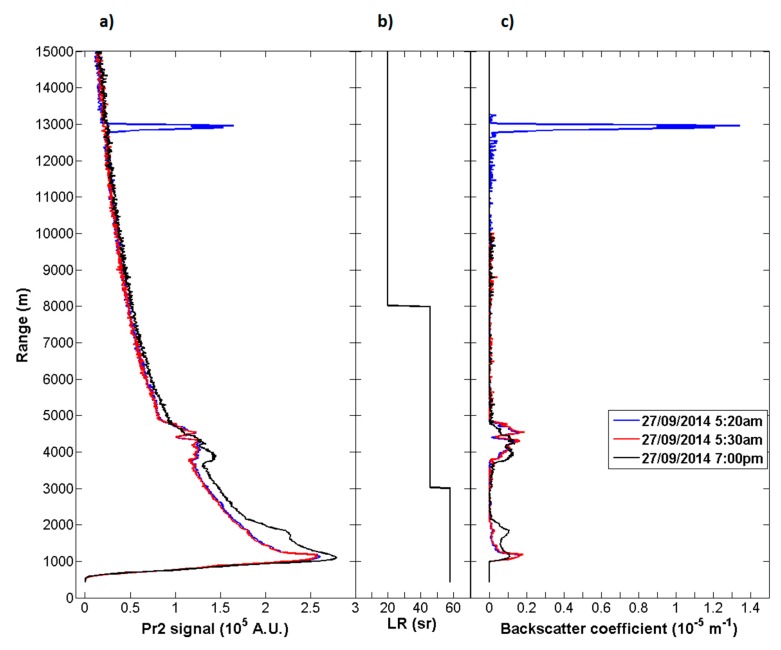
Lidar profiles obtained during the 27 September 2014 at 5:20 UT (blue), 5:30 UT (red) and 19:00 UT (black) presenting cirrus and forest fire aerosol layer respectively around 13 km and 4 km: (**a**) Pr^2^ range corrected signal, (**b**) LIDAR Ratio and (**c**) backscatter coefficient.

A special day presenting aerosol event from American fires and cirrus observations is presented on the Figure 14, focusing on three specific times observations on 27 September 2014, at 5:20 UT, 5:30 UT and 18:10 UT, respectively (Figure 14). Each profile corresponds to 10 minutes integration time with a vertical resolution of 15 m. The Rayleigh fit is applied between 13,500 and 15,100 m, between 7000 and 10,000 m and between 6000 and 6500 m, respectively, corresponding to the molecular reference signal. The aerosol layer is observed between 4 and 5 km with a backscatter coefficient of 5 × 10^−6^ m^−1^ and the cirrus event around 13 km with a more significant signal (3.5 × 10^−5^ m^−1^). Both corrected signals and backscatter coefficients showing close aerosol layer measurements at 5:20 UT and 5:30 UT. At 18:10 UT, the aerosol signal decreases due to the plume dilution (from 5 × 10^−6^ m^−1^ to 2 × 10^−6^ m^−1^). A cirrus event appears only on the first profile, at 5:20 UT, in parallel with the aerosol event. An algorithm to retrieve cirrus clouds scattering ratio and depolarization ratio is under development.

**Figure 15.**
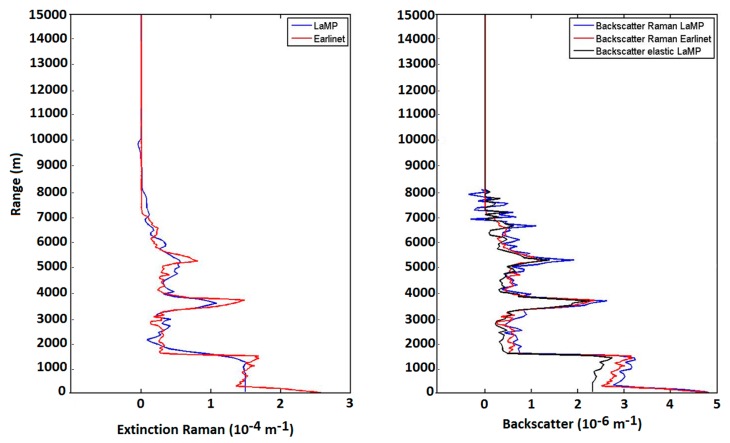
(**Left**): Initial (red) and retrieved (blue) extinction coefficient profile; (**Right)**: Initial (red) and retrieved (black elastic, blue Raman) backscatter coefficient profile.

Based on the equations described in [42] the retrieval of the aerosol extinction coefficient and the LIDAR ratio with Nitrogen Raman channel was also developed for night-time measurements. In the frame of the EARLINET network this algorithm was compared with other algorithms within the network. A LIDAR signal was simulated from a given backscatter coefficient and LIDAR ratio profiles. The method and the results are described in [43]. The algorithm developed was tested on the same data set and the results are presented in the Figure 15. This algorithm is now under implementation for routine processing.

## 7. Conclusions

In this paper we provide a detailed description of the Rayleigh-Mie Raman LIDAR system of Clermont-Ferrand and of the technical motivations for improvements and modifications made on the system. The automation of measurements allows routine continuous measurements with a limited manpower. This methodology can be applied to other LIDAR systems. First measurements of water vapour, cirrus and aerosols profiles demonstrate that this system provides new capabilities in response to the increasing needs for the cycles of these atmospheric components and processes to be studied and survey in a framework of climate evolution.

The continuation of routine measurements in the long term is very important for climate issue and we plan to ensure it. Some additional improvements of the system and data processing are also planned. For example, the improvement of Raman channels could allow us to retrieve aerosol/cirrus profiles without assumption on the LIDAR ratio. The use of independent observation data such as GPS water vapour columns is also envisaged. Finally, the aerosol, cirrus and water vapour profiles on a routine basis open many scientific perspectives in the volcanic plumes characterization, cirrus and water vapour radiative impact and short time variations domains.

## Appendix

### Determination of the Volume Depolarisation Ratio

The principle for determining the volume depolarisation ratio δ^ν^ is explained in [13,14]. The laser polarisation offset angle ϕ is corrected with the half-wave plate. The method consists in comparing the acquired reflected signals P_R_ (−45° + ϕ) and P_R_ (45° + ϕ) (respectively the acquired transmitted signals P_T_ (−45° + ϕ) with P_T_ (+45° + ϕ)), then this angle is adjusted in order to equalize P_R_(±45° + ϕ) (respectively P_T_(±45° + ϕ)) which means that ϕ = 0. In this configuration, the volume depolarisation ratio is given by:

(A1) $$\delta^{\text{v}}=\frac{{\text{P}}_{\text{S}}(0{}^{\circ})}{{\text{P}}_{\text{P}}(0{}^{\circ})}$$

where P_p_ is the incident signal coming from the atmosphere, with a parallel polarisation plane to the incident plane of the PBS cube, and P_s_ is the incident signal with a cross polarisation plane to the incident plane of the PBS cube. This means that the acquired signals P_R_ and P_T_ can be written as:

(A2) $$\begin{array}{l} {\text{P}}_{\text{R}}\left(\theta \right)={\text{V}}_{\text{R}}\cdot {\text{R}}_{\text{S}}\cdot {\text{P}}_{\text{S}}\left(\theta \right)\\ {\text{P}}_{\text{T}}\left(\theta \right)={\text{V}}_{\text{T}}\cdot {\text{T}}_{\text{P}}\cdot {\text{P}}_{\text{P}}\left(\theta \right) \end{array}$$

where V_R_ (respectively V_T_) corresponds to the global transmission factor for the signal reflected (respectively transmitted) after the PBS cube (*i.e.*, transmissions of the optic component after the PBS cube, quantum efficiency of the PMT and amplification on the acquisition channel), T_p_ and R_s_ are respectively the global transmission coefficient of the parallel signal, and the global transmission of the cross signal, between the half-wave plate and the PBS cube. We consider as well that R_p_ = T_s_ = 0 thanks to the polariser plates in front of the PMTs, which suppress the cross-talk of the PBS cube. At ± 45°, we have P_s_(±45°) = P_p_(±45°). So:

(A3) $$\delta^{*}(\pm 45{}^{\circ})=\frac{{\text{P}}_{\text{R}}(\pm 45{}^{\circ})}{{\text{P}}_{\text{T}}(\pm 45{}^{\circ})}=\frac{{\text{V}}_{\text{R}}\cdot {\text{R}}_{\text{S}}}{{\text{V}}_{\text{T}}\cdot {\text{T}}_{\text{P}}}$$

which depends only on the transmission characteristics of the system.

The ± sign means that the geometric average of the + 45° and −45° is used, so:

(A4) $$\delta^{*}(\pm 45{}^{\circ})=\sqrt{\delta^{*}(+45{}^{\circ})\cdot \delta^{*}(-45{}^{\circ})}$$

The volume depolarisation ratio is obtained by replacing P_s_ and P_p_ in Equation (A1) by P_R_ and P_T_ from Equation (A2):

(A5) $$\delta^{v}=\frac{{\text{P}}_{\text{R}}}{{\text{P}}_{\text{T}}}\cdot \frac{{\text{T}}_{\text{P}}\cdot {\text{V}}_{\text{T}}}{{\text{R}}_{\text{S}}\cdot {\text{V}}_{\text{R}}}=\frac{\delta^{*}(0{}^{\circ})}{\delta^{*}(\pm 45{}^{\circ})}$$

which corresponds to the Equation (2). The relative transmission factor V*, which is the ratio V_R_/V_T_, is given by the Equation (A3) and (A4).
